# Fabrication of TiVO_4_ photoelectrode for photoelectrochemical application†

**DOI:** 10.1039/d2ra05894d

**Published:** 2022-12-02

**Authors:** Manal Alruwaili, Anurag Roy, Srijita Nundy, Asif Ali Tahir

**Affiliations:** Environment and Sustainability Institute, Faculty of Environment, Science and Economy, University of Exeter Penryn TR10 9FE UK ma942@exeter.ac.uk a.tahir@exeter.ac.uk; Physics Department, Faculty of Science, Jouf University PO Box 2014 Sakaka 42421 Saudi Arabia

## Abstract

Photoelectrochemical (PEC) water splitting is one of the promising, environmentally friendly, carbon emission-free strategies for the cost-effective production of hydrogen. The interest in developing effective approaches for solar-to-hydrogen production with stable and visible light active semiconductors directed many researchers to develop stable and efficient materials. For the first time, a nanostructured TiVO_4_ photoanode was fabricated at a substrate temperature of 250 °C and further annealed at 600 °C using the spray pyrolysis technique and it obtained an optical band gap of ∼2.18 eV. The photoanode underwent photoelectrochemical testing, where it exhibited a high photocurrent density of 0.080 mA cm^−2^ at 1.23 V (*vs.* reversible hydrogen electrode), which can be stable up to 110 min. Further, various physicochemical characterizations were employed to understand the phase purity and thin film growth mechanism. A systematic substrate and annealed temperatures were monitored during the fabrication process. The transmission electron microscopy (TEM) studies revealed agglomeration of TiVO_4_ nanoparticles with an average size of ∼100 nm accompanying dendritic orientation at the outer edge. This study envisages the design and development of a novel photocatalyst for water splitting under visible light irradiation, an ideal route to a cost-effective, large-scale, sustainable route for hydrogen production.

## Abstract 2

TiVO_4_ photoanode was prepared using the spray pyrolysis technique and further employed for photoelectrochemical water splitting to produce hydrogen.

## Graphical abstract

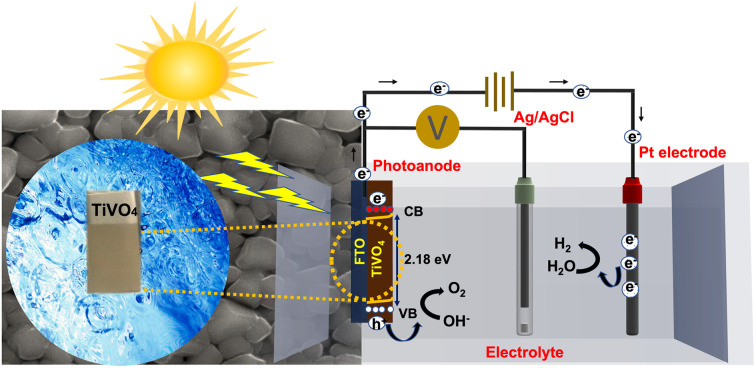

## 1. Introduction

The most incredible mission of the current scientific generation is to address the considerable energy demands.^1^ Researchers are devoting their efforts to searching for secure, safe, and sustainable energy options to cope with expected nonrenewable energy shortages and eliminate environmental pollutants. Solar energy is expected to be the leading and safe option that can solve the energy crisis, targeted explicitly through improvements to ensure access to reliable, sustainable, affordable and modern energy. An understanding of renewable-energy solutions for water splitting, which is the process of hydrogen production by direct water decomposition, heavily depends on further developments in cost-effectiveness and environmental technologies. The photoelectrochemical (PEC) water splitting is considered one of the most promising H_2_ generation methods with required potential (Δ*G*^°^ = 237 kJ mol^−1^), due to its use of the unlimited energy source of solar light without producing any CO_2_.^2–5^ Since 1972, after the pioneering work by Fujishima and Honda using TiO_2_ as a photocatalyst in a water splitting system.^6^ To achieve high performance of PEC materials, suitable band gap, diffusion length, bulk defects and surface recombination, *i.e.* accessible surface area, porosity, pore volume and high crystallinity, are critical parameters to affect the overall PEC performance.^7^ Particularly, porosity and pore fall into the requirements for low diffusion length materials, such as some metal oxides.^8–10^

Fe_2_O_3_,^11^ Co_3_O_4_,^12^ TiO_2_,^13^ MoO_*x*_ ^14^ and VO_2_ ^15^ are materials that have been used widely in PEC applications, but some drawbacks suppress their work, such as fast charge carriers recombination and low charge transport. In addition, these semiconductors' band edge potentials are incompatible with the redox potentials for some specific photocatalytic redox reactions. Hence, if these bimetallic semiconductors can be combined with other novel photoactive materials such as NiO,^16^ ZnO,^17^ CdO (ref. 18) *etc.*, to construct heterojunctions such as Co_3_O_4_/TiO_2_,^19^ Fe_2_O_3_/Au/TiO_2_,^20^ ZnO/Fe_2_O_3_,^21^ Fe_2_O_3_/CdO,^22^ MoO_*x*_/GaP ^23^ and ZnO/NiO ^24^*etc.* with proper band alignment, they could serve as efficient PEC material as compared to single-phase metal oxide. It can be combined with other dopants to extend those materials' spectral response towards visible light.^25–27^

Recently, bimetallic oxide materials (BiVO_4_,^28^ Fe_*x*_V_*x*_O_4_,^29^ Cu_*x*_V_*x*_O_*x*_ ^30^ have become promising photoelectrode due to their tuned bandgap, suitable for PEC water splitting. They have shown an effective usage of solar irradiation owing to their synergic absorption in the visible and ultraviolet (UV) regions.^23,31^ Numerous pioneering reports highlighted the superior performance of TiV systems as composites VO_*x*_/TiO_2_ or as doping compared to TiVO_4_ – bimetal oxide on their plausible photocatalysis application.^32,33^ Samdarshi *et al.* (2010) evaluated the effect of silver on the titanium vanadium mixed metal (Ag/TiV) oxides.^34^ They observed that the system could absorb a large portion of the visible spectrum, where favourable electron transfer could be experienced anatase-rutile mixed phase where silver dominated through its scavenging action to reduce hole recombination. On the other hand, the Ag-doped TiV films were developed by the sol–gel technique, increasing antibacterial activity when exposed to visible light.^35^ The visible transmittance can be substantially improved, resulting in a higher photocatalytic property under visible light irradiation for the Ti–V system.^36^ Alternatively, an efficient way to use the majority of sunlight could be achieved using nanocomposite semiconductors such as TiO_2_–V_2_O_5_.^37^ Li *et al.* (2016) investigated V^4+^ ion doping to decorate TiO_2_ nanocrystals, which tailored energy level alingment of TiO_2_ nanostructure by shortening the electronic transfer lifetime to 34.7% compared to only TiO_2_.^38^ By contrast, no reports have gone on to utilize TiV systems in PEC applications. Extending these advanced characteristics to PEC systems will undoubtedly bring breakthroughs in PEC-driven photo-to-energy conversions. The main challenge now is to design optical paths to achieve high light absorption with high activity, durability, and selectivity during the PEC analysis.

Single-phase TiVO_4_ is still not explored much and received little attention, which can promise effective PEC characteristics reported by various TiV systems. Comparing TiVO_4_ to TiO_2_, its interfacial structure is superior for higher electron diffusion during high-temperature fabrication, creating a low impedance contact possessing high optical transparency and providing long-term stability. By comparing the requirements of novel PEC materials, TiVO_4_ can be achieved with similar small-radius cations, which are directed to narrow band gap and have transition metals as the cation component, which is earth-abundant, cost-effective, and low-toxicity, benefiting wide usage in the future.

This work synthesized a phase pure TiVO_4_ as a photoanode using the scalable spray pyrolysis technique. The photoanode produced by spray pyrolysis has a large surface area of substrate coverage potential, a cost-effective method and homogeneity of mass synthesis. The influence of the thin films' substrate and annealing temperatures is further studied and exhibited remarkable hierarchical structures. Furthermore, the spray pyrolyzed TiVO_4_ photoanode significantly resulted in a narrower visible band gap with a high photocurrent, further employed for the PEC water splitting application. The following sections thoroughly investigated and discussed an underlying phenomenon for this superior performance.

## 2. Materials and methods

### 2.1. Fabrication of TiVO_4_ thin film *via* spray pyrolysis

All chemicals used for the fabrication of TiVO_4_ were purchased from Merck Life Science Products (UK) and used without further purification.

**Fig. 1:**
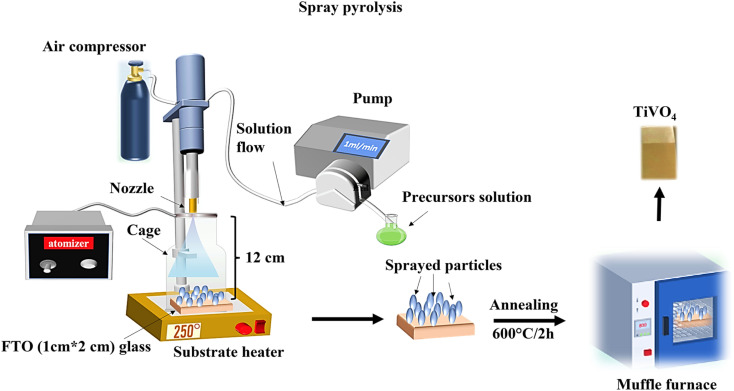
Schematic representation of a TiVO_4_ photoanode fabrication process using spray pyrolysis technique under optimised conditions.

TiVO_4_ photoanodes were prepared *via* spray pyrolysis. The solution was prepared by dissolving vanadium acetylacetonate, and titanium isopropoxide in 15 mL of ethanol with a total concentration of 0.05 M. Next, 0.05 mL of trifluoroacetate acid (99%) was added to the mixture. It was kept under stirring for 2 h to get a clear homogenous solution. The solution was employed in the spray pyrolysis system, which comprises a syringe pump (New Era Pump system NE-1000), an attached vortex, and an ultrasonic atomizer (Sonozap) assisted with a nozzle of 1 mm (diameter). In this process, the prepared spray droplets are transported to the substrate through an atomizing nozzle. The evaporation of the solvent within the spray droplets leads to the formation of a uniform coating on the substrate. The parameters of the spray pyrolysis were maintained as follows, 10 mL of solution was sprayed on the 1 cm × 1 cm cleaned fluorine-doped tin oxide (FTO) glasses kept at a distance of 12 cm from the nozzle head at a flow rate of 1 mL min^−1^, which is assisted with the compressed air (4 L min^−1^). The deposition on the FTO glass was performed under various temperatures from 150 °C to 300 °C at an interval of 50 °C, referred to as substrate temperatures. Once the spray FTO finished, substrates were then taken for annealing at different temperatures from 500 °C to 650 °C, at an interval of 50 °C, for 2 h in a muffle furnace. The TiVO_4_-coated FTO substrates were allowed to cool to room temperature before being taken for further analysis. Fig. 1 represents a schematic illustration of a TiVO_4_ photoanode fabrication process using spray pyrolysis technique under optimised conditions.

### 2.2 Materials characterisations

Thin film microstructure analysis was performed using the TESCAN VEGA3 scanning electron microscopy (SEM) equipped with the energy dispersive spectroscopy (EDS) from Oxford Instruments. The structure and phases of the TiVO_4_ thin films were characterised using a Bruker D8 X-ray diffractometer (XRD) assisted with a monochromatic CuK_α_ (*λ* = 0.154 nm) radiation. XPS Analysis was performed using a Thermo NEXSA XPS fitted with a monochromated Al K_α_ X-ray source (1486.7 eV). All sample data was recorded at a pressure below 10^−8^ Torr, and a room temperature of 294 K. Data was analysed using CasaXPS v2.3.20PR1.0 and calibrated with C 1s peak at 284.8 eV. Corresponding transmission electron microscopy (HR-TEM) analysis was carried out using JEOL JEM-2100F TEM (200 kV). The absorption and transmission spectra of the thin film were executed by PerkinElmer's UV-VIS-NIR UV-3600 Plus spectrophotometer.

TiVO_4_ photoanode was employed for PEC studies, which were conducted utilising the Metrohm Autolab (PGSTAT302N) workstation consisting of three-electrode compartments. Where a platinum wire was used as the counter electrode, a 3 M aqueous solution of Ag/AgCl in KCl was considered the reference electrode, and 1 M aqueous solution of NaOH (pH of 13.6) was employed as the electrolyte for the electrochemical testing. The light intensity was simulated to achieve 1 SUN condition (100 mW cm^−2^) from Newport, consisting of a 300 W xenon lamp with AM 1.5 filter and 420 nm cut-off filter to remove the UV part of the sunlight. The photoanode's voltages (potential *vs.* Ag/AgCl) were recorded at a scan rate of 0.01 V s^−1^ from negative to a positive potential direction (−0.3 V and +0.8) under light, dark and chopping conditions. All potentials were then converted to reversible hydrogen electrode (RHE) potential according to the Nernst eqn (1) as,

1 *E*_RHE_ = *E*_Ag/AgCl_ + 0.0591(pH) + 0.1976 V

where the pH of the electrolyte was maintained at 13.6. Furthermore, the Mott–Schottky relationship was employed to determine the photoanode's flat band potential (*V*_fb_).

## 3. Results and discussions

### 3.1. Structural and optical analysis

**Fig. 2:**
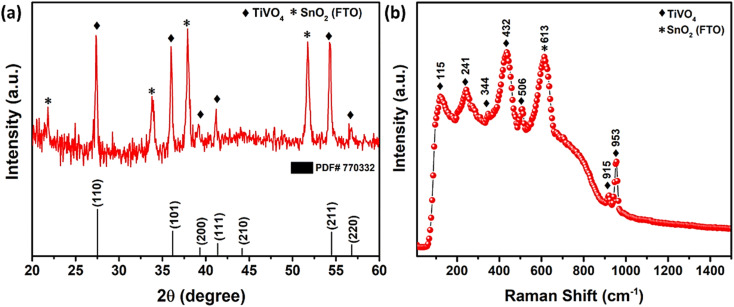
(a) XRD pattern, (b) Raman spectrum of spray pyrolyzed TiVO_4_ photoanode deposited on FTO glass.

The XRD pattern of synthesized TiVO_4_ photoanode exhibits single-phase material at 600 °C, as shown in Fig. 2a. The diffraction peaks correspond to 27.5°, 41.3° and 54.4°, representing the tetragonal lattice planes of (110), (111) and (211) for TiVO_4_ structure. These results also corroborate with the JCPDS file 01-770332.^34^ Besides, with almost similar intensity, the distinct peaks indicate FTO-glass substrate, and XRD confirms TiVO_4_ thin film formation. No additional peaks of either TiO_2_ or VO_2_ hence admittedly manifested pure phase of TiVO_4_ tetragonal structure. However, the XRD analysis was also performed at different substrate temperatures during the spray pyrolysis and further different annealing temperatures (Fig. S1, ESI†) to optimize the phase pure TiVO_4_ thin film development. The optimized substrate and annealed temperatures were found to be 250 °C and 600 °C, respectively. Below these temperatures, either the tetragonal TiVO_4_ phase does not entirely form, or the film deposition was not homogeneously covering the FTO surface; as a result, the XRD patterns revealed dominated peaks attributed to the FTO glass.

Fig. 2b revealed the Raman spectrum of the optimized TiVO_4_ thin film, confirming the successful formation of tetragonal TiVO_4_ on the FTO glass. The stretching modes of V–O combining Ti–O and V–O occurred at higher wavenumber bands at ∼915 and 953 cm^−1^.^39^ The bending and stretching modes appeared in the 400–600 cm^−1^ region. Other <400 cm^−1^ bands were external modes from the lattice, translational, and vibrational motions. Noticeably, Raman analysis of TiVO_4_ (tetragonal) is rare; however, the concerning result has been compared with the CrVO_4_ (orthorhombic) and FeVO_4_ (triclinic) Raman bands relevant to TiVO_4_.^40^ It is anticipated that due to the difference in electronegativity of these metal, all the strongest peak of TiVO_4_ exhibits a slightly lower intensity compared to CrVO_4_ and FeVO_4_. Also, because of its tetragonal and nondegenerate vibrations, the Raman bands of TiVO_4_ were between CrVO_4_ and FeVO_4_. A peak at ∼613 cm^−1^ corresponds to the A_1g_ vibrational mode of F-doped SnO_2_ originating from the FTO glass.^41^

**Fig. 3:**
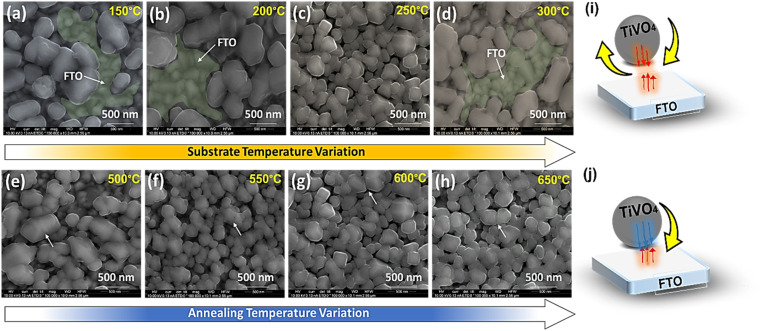
SEM microstructural images of TiVO_4_ photoanode at different substrate temperatures such as (a) 150 °C, (b) 200 °C, (c) 250 °C, (d) 300 °C where annealing temperature was fixed at 600 °C, and at different annealing temperatures such as (e) 500 °C (f) 550 °C (g) 600 °C (h) 650 °C, where the substrate temperature was fixed at 250 °C, respectively, schematic illustration of the thermophoretic effect of the (i) high substrate temperature of 300 °C and (j) optimized temperature of 250 °C on the particles deposition.

Fig. 3a–d reveals SEM microstructural images of TiVO_4_ photoanode developed at different substrate temperatures of 150 °C, 200 °C, 250 °C and 300 °C, where the annealing temperature was maintained at 600 °C. The substrate temperature profoundly impacts the morphology and performance.^42–45^ At lower substrate temperatures of 150 °C to 200 °C, the spray droplets splash directly onto the substrate (FTO) and decompose without evaporating the solvent in (Fig. 3a and b).^46^ As a result, the particles agglomerated with an average size of ∼700 nm, leaving bare FTO as shown in SEM (green coloured). This is due to the rapid droplet flux reaching the substrate without a complete nucleation growth process, leading to rough films and an ununiform distribution of the material on FTO glass. Whereas at the substrate temperature of 250 °C, the solvent will leave before reaching the substrate, and a solid precipitate of precursor will fall on the substrate providing a homogenous coating. As a result, growth is uniform, and the bead-like structure with reduced particle size narrowed down to 300 nm, securing better coverage and homogeneous deposition across the FTO glass, as shown in Fig. 3c. However, at 300 °C substrate temperature, the decomposition of precursor starts before reaching the substrate, resulting in less adhesion to the substrate and giving powder particles on the surface, which peel off during the annealing process leaving bare FTO as shown in (Fig. 3d) (green coloured).^47,48^ Based on the morphology observed by SEM; it is clear that the optimum substrate spray temperature for uniform and smooth films was found to be 250 °C.

Fig. 3e–h displays the impact of the various annealing temperatures from 500 °C to 650 °C on the films deposited at a substrate temperature of 250 °C. The images from Fig. 3e–h show that the only significant difference in morphology of the films is observed on those annealed at 500 °C. The morphology at this annealing temperature shows the wide distribution of the particle's size ranging from 600 to 150 nm, whereas the morphology of all those films annealed at 550 to 650 °C shows almost similar size distribution and more uniform growth. As well as Fig. 3e and f indicate that particle boundaries are agglomerated. While once the annealed temperature rises to ≥600 °C, the particles are comparatively distinct. The increase in porosity found in the films can be attributed to increased temperature, and the film becomes denser without cracks at higher temperatures. The thermophoretic effect during the high and optimized substrate temperatures of the TiVO_4_ particle deposition was explained through a schematic, as shown in Fig. 3i and j, respectively. The substrate temperatures determine the rate of particle formation and their homogenous distribution. The growth of particles and concerning coverage maximized at 250 °C, and calcination temperature governs the phase purity and morphology of the fabricated particles. At higher substrate temperatures, particles' supersaturation rate and mobility were expected to be high and therefore yielded higher nucleation rates. In contrast, higher mobility facilitates nucleation collision and different growth rates of individual particles. Hence, by understanding the substrate and annealing temperature effect on morphology and homogeneity of the TiVO_4_ photoanode, the optimised spray annealed temperatures are 250 °C, and 600 °C were taken for further analyses.

**Fig. 4:**
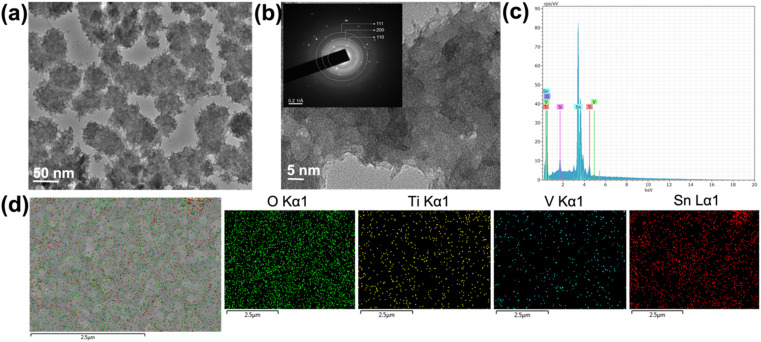
(a) TEM bright field image, (b) corresponding HRTEM and SAED images (inset), (c) EDS spectrum and (d) corresponding element colour mapping of the TiVO_4_ photoanode.

Furthermore, the high-resolution TEM was conducted to get detailed information and understand the nanostructured morphology and crystallinity of the TiVO_4_. The TEM images shown in Fig. 4a display scattered nanoparticle assemblies of TiVO_4_, leading to an average diameter of ∼100 nm. The TiVO_4_ particles exhibit porous features, with some dendrites on their outer edge. Nanoparticles have appeared to form some extent of designed agglomeration. Fig. 4b represents corresponding high-resolution TEM images and selected area diffraction patterns (HR-TEM) images in the inset, indicating the tetragonal lattice fringes match well with the (111), (200) and (110) crystal planes of TiVO_4_, indicating the presence of Ti, V in the composite form. T EDS spectrum was measured to further confirm the doping of vanadium ions into the TiO_2_ lattice, as shown in Fig. 4c and S2, ESI.† The spectrum demonstrates the distinct peaks corresponding to Ti (K_α1_) and V (K_α1_) with a ratio of Ti : V as 1 : 1 (16.4% and 16.0%), providing the composite to be formed as TiVO_4_. Furthermore, elemental color mapping was also carried out, aimed to be a suitable technique to validate the presence of TiVO_4_ onto the FTO glass as shown for the TiVO_4_ thin film as shown in Fig. 4d. It shows the coexistence of Ti, V, Sn and O belonging to TiVO_4_. This result further confirms the deposition of TiVO_4_ onto the FTO glass.

**Fig. 5:**
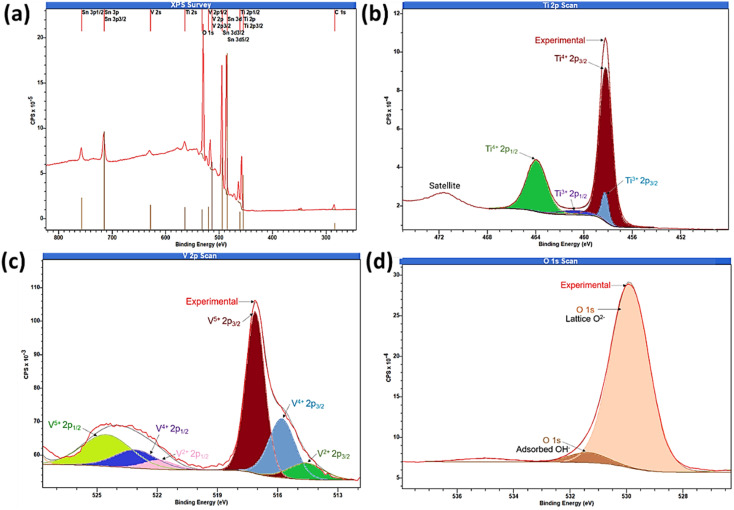
X-ray photoelectron spectroscopy (a) survey spectrum and core level spectrum of (b) Ti 2p, (c) V 2p and (d) O 1s regions of TiVO_4_ thin film deposited on a FTO glass.

As photoelectrochemical activities are surface-driven phenomena, to understand the oxidation state of the elements present on the outermost surface, the surface composition of the sample was analysed by XPS integrated peak area analysis as shown in Fig. 5. The XPS survey spectra of TiVO_4_ photoanode indicates the presence of Ti 2p, V 2p and O 1s, without any impurity and successfully deposited on the FTO glass, indicated by Sn 3d_3/2_ peak as displayed in Fig. 5a. The XPS peaks at 458.7 eV and 464.5 eV corresponding to the binding energies of the Ti 2p_3/2_ and 2p_1/2_ (Fig. 5b). The spin–orbit splitting energy of 5.8 eV is the characteristic of Ti^4+^ oxidation state present in the TiVO_4_ structure.^49^ In addition, the Ti 2p core level spectra can be fitted into Ti^3+^ peaks at ∼457.8 eV and ∼461.6 eV corresponding to Ti^3+^ 2p_3/2_ and Ti^3+^ 2p_1/2_, originating due to the partial reduction of Ti^4+^ state by the generation of oxygen vacancies in the reducing atmosphere. It is reported that Ti^3+^ species and/or oxygen vacancies may significantly improve the observed electronic conductivity of the material.^50^ However, compared to the Ti^4+^ oxidation state, the Ti^3+^ oxidation state is negligible.

There are strong hybridization between V 2p and O 1s states and thus exposed various oxidation states of V 2p during TiVO_4_ formation. The peaks at 516.3 and 517.2 eV correspond to the binding energies of V 2p_3/2_ for V^4+^ and V^5+^, respectively, as shown in Fig. 5c. Besides, the V 2p_1/2_ peaks are positioned at 524.4, and 523.1 eV corresponding to V^5+^ and V^4+^.^51^ V^5+^ might result from the surface oxidation of the samples in the air, which is common in vanadium-based samples.^52^ The observed broadening of the V 2p_3/2_, the linewidth is due to the decrease of the oxidation state. As a result, V^2+^ peaks were also observed.^52^ However, the formation of VO has the oxidation state V^2+^, which is not reported in any previous study. Therefore we cannot unambiguously assign the third component to a vanadium oxidation state. The overlapped mixed-valence oxidation states of V^5+^ and V^4+^ are usually observed in ternary vanadium-based oxides.^53^ Such overlapping peaks contain mixed valences of V^5+^ and V^4+^. Also, the V_2p_ binding energies of TiVO_4_ were lower than the bare V_2_O_5_ and VO_3_ due to the ternary oxide composition. Eventually, the pristine of TiVO_4_ show a ratio V/O ∼0.27, in agreement with the ratio found in the TiVO_4_ (0.25); thus, it is expected that only V^4+^ is present in the sample. The existence of V^5+^ species in the TiVO_4_ thin film suggests that during the chemical synthesis, possible interactions of between the Ti–V sol produce a partial shift to higher binding energy in concordance with the V^5+^ species.

Further, two peaks for oxygen (O 1s) were observed, one for O^2−^ in the metal oxide as interaction strength of the V=O bonds, and O=V–O–V^4+^(V^5+^)–O–Ti^4+^ to form TiVO_4_. While the other one at 532.2 eV characteristics of chemisorbed oxygen on transition-metal as shown in Fig. 5d.^54^ The surface atomic percentages calculated from the XPS data were Ti 2p ∼9%, V 2p ∼8.2%, O 1s ∼36% and Sn 3d ∼4%. The elemental composition and distribution are corroborated with the XRD, EDS and XPS measurements. These results further proved that the nanostructures of TiVO_4_ had been successfully grown on the surface of FTO glass.

**Fig. 6:**
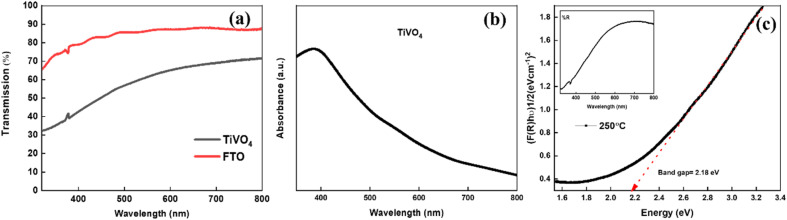
(a) Transmittance spectra of TiVO_4_ photoanode fabricated at an optimized substrate temperature 250 °C and calcined at 600 °C, compared with FTO, corresponding (b) absorption (c) reflectance spectra concerning the Kubelka–Monk plot, respectively.

Fig. 6a exhibits the transmittance spectra of the TiVO_4_ photoanode and compares it with the FTO glass. The FTO glass shows an average transmittance of ∼80%, whereas the TiVO_4_ thin film exhibits slightly less steady transmittance to ∼50. Reduction in transmittance indicates the formation of a thin film on FTO glass. During the spray pyrolysis process, the thin film retained semi-transparent behaviour for all the samples. This is also evident from the thin film's colour, which visually appears light yellow. During the spray pyrolysis process, the thin film retained semi-transparent behaviour for all the samples. This is also evident from the thin film's colour, which visually appears light yellow. Fig. 6b shows the maximum absorption edge of TiVO_4_ ∼ 400 nm, which shows an exponential decrease towards the visible wavelength region.

Moreover, the absorption edge of the composite has been extended to the visible region up to 600 nm due to incorporating V^4+^ in the TiO_2_ structure, which could help harvest the UV and visible components of the solar radiation. The optical bandgap estimation was calculated using reflectance measurement from the Kubelka Munk equation, as shown in Fig. 6c. The band gap of TiVO_4_ is estimated to be ∼2.18 eV. TiVO_4_ bandgap was narrower than TiO_2_, where V^4+^ incorporation plays a crucial role. This may be because the ionic radii of V^4+^ are 0.72 Å, which is close to the Ti^4+^ ionic radii (0.74 Å), which signifies interstitial doping of V^4+^ within TiO_2_ lattice TiO_2_ enables tuning its light-absorbing capacity. Narrowing the wide bandgap indicates TiVO_4_ could be used as an anodic electrode for water-splitting applications.

### 3.2. Photoelectrochemical analysis

**Fig. 7:**
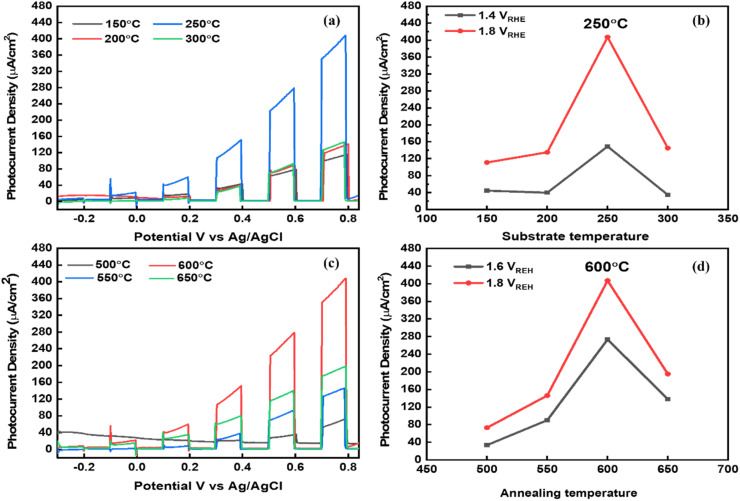
Linear sweep voltammetry (LSV of current density – potential *vs.* Ag/AgCl) plots under 100 mW cm^−2^ chopped light and dark illumination at a scan rate of 0.2 mV s^−1^ in 1 M NaOH electrolyte (pH = 13.6) showing (a and b) effect of substrate temperatures and (c and d) annealing temperatures on the PEC performance.

Considering the optical analysis, nanostructure, and phase pure properties, the fabricated TiVO_4_ photoanodes were employed for PEC investigation with varied substrate and annealing temperatures. The linear sweep voltage (LSV) plot (Fig. 7a), *i.e.*, photocurrent (μA) *vs.* potential (*vs.* Ag/AgCl), was performed at a scan rate of 1 mV s^−1^ of the TiVO_4_ as a working electrode was recorded under chopped conditions (light and dark with chopping at an interval of 0.2 V *vs.* Ag/AgCl) for various substrate temperatures fabricated films. Among the various substrate temperatures, the photoanode developed at 250 °C recorded the highest photocurrent of 400 μA at 0.8 V *vs.* Ag/AgCl (1.8 V *vs.* RHE), which is much higher than the other samples sprayed at 150 °C (108 μA), 200 °C (135 μA) and 300 °C (145 μA). Fig. 7b indicates the trend of photocurrent obtained by the TiVO_4_ photoanode developed at various substrate temperatures at 1.4 and 1.8 V *vs.* RHE. The photocurrent slightly increases with an increase in temperature from 150 to 200 °C, *i.e.*, from 108 to 135 μA. Intermediate adhesion and incomplete phase development with low coverage of the particles on FTO glass could have resulted in low photocurrent.^55^ At 250 °C, it drastically enhanced to 400 μA. This phenomenon is mainly attributed to the sufficient temperature required to evaporate the organic materials, forming the pure phase material. At an optimised substrate temperature, maximum nucleation density and proper atomic diffusion can be achieved, resulting in the material's complete growth.^56,57^ However, at 300 °C, the photocurrent decreases significantly beyond this temperature to 140 μA. This is ascribed to the agglomeration and decomposition of the materials at higher temperatures and corroborates with the SEM data. The overall trend of photocurrent generation for different substrate temperatures was followed as 150 °C < 200 °C ∼250 °C > 300 °C.

Next, to evaluate the effect of different annealing temperatures on the TiVO_4_ photoanodes, the LSV photocurrent transients were obtained, as shown in Fig. 7c. Here, the substrate temperature was maintained at the optimized temperature, *i.e.*, 250 °C. A significant enhancement in the photocurrent is noticed for the thin film annealed at 600 °C. The photocurrent seemed to increase monotonically from 73 to 400 μA with an increase in annealing temperature from 500 to 600 °C. This was due to better coverage on the substrate, uniformity of the thin film as observed by SEM, and improved crystallinity improvement as confirmed by XRD. The overall trend of photocurrent generation for different substrate temperatures was followed as 500 °C < 550 °C < 600 °C > 650 °C. The trend for photocurrent achievement at different annealing temperatures at 1.4 and 1.8 V *vs.* RHE was further described in Fig. 7d. Nevertheless, higher annealing temperature (650 °C) displays a drop in the photocurrent to 196 μA, primarily due to the increasing its overall resistivity of films which may be due to the sintering of particles to the increased size and reduce the surface area.^58^

**Fig. 8:**
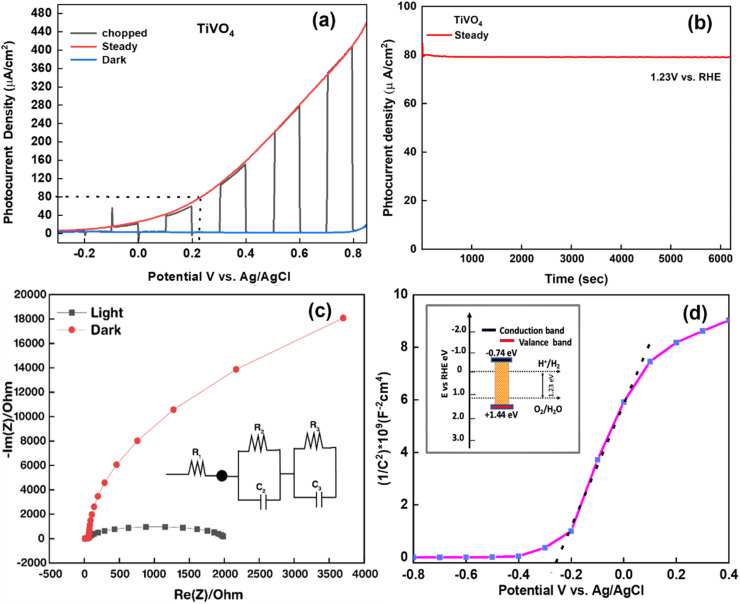
(a) LSV curves of optimized TiVO_4_ film under light–dark-chopped conditions (b) photocurrent stability plot under continuous 1 SUN illumination for 12 h (c) electrochemical impedance spectroscopy data obtained for TiVO_4_ photoanode under dark–light condition (circuit diagram shown in the inset) (d) Mott Schottky data are showing the flat band potentials.

The linear sweep voltammogram (LSV) of the optimised TiVO_4_ photoanode was plotted in Fig. 8a and measured under dark and illumination conditions with an onset potential of ∼0.8 V (*vs.* RHE). Herein, the photocurrent was significantly increased over the positive potential scan range, which indicates that TiVO_4_ is working as an effective photoanode material. At the bias potential of 1.8 V *vs.* RHE, photocurrent density was measured to be the highest value of 400 μA cm^−2^. Also, the stability of optimised TiVO_4_ was investigated using the chronoamperometry technique by keeping the film under illumination at a maintained applied bias potential of 0.23 V *vs.* Ag/AgCl (1.23 V *vs.* RHE), representing the required PEC water potential for 6000 s Fig. 8b. Steady-state of photocurrent density was extrapolated over tested time without degradation of the total of photocurrent density. Furthermore, to investigate the charge transport kinetics of the TiVO_4_, electrochemical impedance measurements were carried out under both dark and 1 SUN illumination (100 mW cm^−2^). The Nyquist plots of electrochemical impedance spectra of obtained data are shown in Fig. 8c, with an equivalent circuit (*R*_1_ + *R*_2_/*C*_2_ + *R*_3_/*C*_3_) in the inset used to evaluate resistance values. *R*_1_ represents the total solution resistances of the circuit among FTO, titanium vanadate film and connecting wires, *R*_2_ is a resistance that arises from charge transfer at the electrode/electrolyte interface, and *R*_3_ represents the resistance of charge transport of the bulk TiVO_4_ film.^59^ Simultaneously, *C*_2_ and *C*_3_ are ascribed to the bulk material's capacitance and the associated surface states. Under light conditions, resistance values of *R*_1_, *R*_2_ and *R*_3_ were found to be 12.41 Ω, 1983 Ω and 113 Ω, respectively, indicating the facile charge transfer of the obtained film and thus promoting PEC activity.

The Mott–Schottky calculation was carried out to determine the space-charge capacitance over potential scan ranges and the flat band potential of the optimized photoanode. The Mott–Schottky plot elucidated the n-type performance of TiVO_4_ film, as shown in Fig. 8d. Flat band potential was estimated to be −0.26 V *vs.* Ag/AgCl and used in the Mott–Schottky eqn (2) to calculate the concentration of the dopants (*N*_D_)2
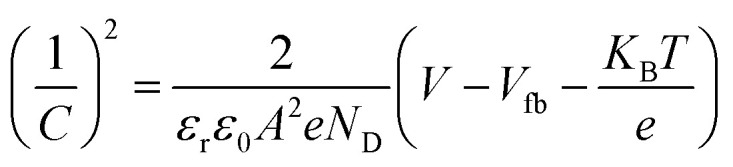
where *C* is space-charge capacitance, *ε*_0_ is the permittivity of vacuum, *ε*_r_ is the relative permittivity of a material, *A* is the area of the film, *N*_D_ is the carrier concentration, *K*_B_ is Boltzmann constant, *T* is temperature, *e* is the electronic charge, *V* is applied potential and *V*_fb_ is the flat band potential which is estimated through linear fit in Mott–Schottky plot. The slope was measured to be 5.6 × 10^9^, and the carrier density was calculated to be 7.7 × 10^20^ cm^−3^. The two bands' positions of the valence band (+1.44 V *vs.* RHE) and conduction band (−0.74 V *vs.* RHE) illustrated in Fig. 8d cover the required redox potentials to drive solar water splitting through the system. Estimated flat band potential refers to the conduction band of the film, as shown in the inset. Finally, this data may elucidate that the composition of TiVO_4_ capable of producing hydrogen energy from the PEC water-splitting process.

In order to explain the overall water splitting in a PEC with an n-type semiconductor TiVO_4_ photoanode, hydrogen is generated at the cathode while oxygen is evolved at the photoanode surface. Thus, the reaction products can be collected in separate chambers. In PEC water splitting, similar to the basic principle of photoanode, *i.e.*, on light irradiation, photoexcitation of the charge carriers followed by their migration to the photoanode surface take place. These photo-generated electrons (e^−^) and holes (h^+^) that migrate to the surface of the photocatalyst without recombination can produce gaseous hydrogen and oxygen, respectively, by reducing and/or oxidizing the water molecules, respectively adsorbed on the photoanode surface. The photo-oxidation of H_2_O by the holes in the valence band causes a surface back reaction which demerits the H_2_ evolution rate.

In this work, the narrow band gap of the obtained film at ∼2.18 eV is suitable to absorb a wide range of incident visible light and thus form electron–hole pairs. The generated charge carries participation into the electrolyte with the species of O_2_ and OH˙ to trigger redox reactions. In brief, photo-generated electrons on the photoanode travel towards FTO, drifting to the cathode surface to carry out the reduction process (H_2_ evolution).^60^ Most importantly, some energy losses may occur due to recombining charge carriers, resulting in low photocurrent density. Next, photo-generated holes remain on the photoanode and usually experience slow kinetics at the interface between photoanode/electrolyte, resulting in hole accumulation, causing charge recombination, thus initiating oxygen evolution. The photoanode of the titanium vanadate film has shown enhanced PEC activity with lower charge transfer resistance observed from the impedance data than in the binary oxide of TiO_2_ and VO_2_ films.^6,15^

In addition, the higher photocurrent density of TiVO_4_ lead to a promising alternative for the PEC application in future, and the observed data has been compared with the other reported materials in Table 1. Impressive photocurrent density and stability tested time were observed for the TiVO_4_ photoanode compared with the other reported thin films developed *via* spray pyrolysis.

**Table 1:** Comparative study of TiVO_4_ photoanode with the latest developed thin films *via* spray pyrolysis for PEC study

Serial no.	Sample	Fabrication/deposition technique	Highest photocurrent (mA cm^−2^) (at 1.23 V *vs.* RHE)	Stability tested time (h)	Reference
1	LaFeO_3_	Spray pyrolysis	0.0180	21	61
2	Bi_2_WO_6_	Spray pyrolysis	0.042	—	62
3	BiFeO_3_	Spray pyrolysis	0.012	—	63
4	ZnFe_2_O_4_	Spray pyrolysis	0.130	1.37	64
5	TiVO_4_	Spray pyrolysis	0.080	1.66	This study

Although significant improvements have been achieved in the construction of highly efficient ternary TiVO_4_ PEC materials, the recombination rate of photo-generated charge carriers for TiVO_4_-based photocatalysts is still considerably high, accounting for poor reduction ability in the photoexcited electrons at a low potential of the conduction band edge, which is efficiently quenched by defects and holes. Morphological engineering could improve the PEC activity of TiVO_4_, regulating the crystal structure, particle size, and surface area leading to a large-scale preparation, which would enormously improve the separation efficiency of the photo-generated charge carriers. Further, TiVO_4_ could pave the way as a promising not only for PEC but also for smart coating and energy conversion.

## 4. Conclusion

This work describes the development of single-phase tetragonal nanostructure TiVO_4_, which is not explored as a PEC material. TiVO_4_ photoanode on FTO glass was fabricated using the scalable spray pyrolysis technique, a simple and efficient synthesis route showing a semi-transparent characteristic with a band gap of ∼2.18 eV. XRD, Raman and XPS spectroscopic analysis confirms the formation of tetragonal phase pure TiVO_4_ on an FTO glass. The development of TiVO_4_ photoanode was studied for various substrate and annealing temperatures. The SEM microstructure analyses revealed the temperature effect of the TiVO_4_ photoanode development. The optimized substrate temperature was found at 250 °C; the annealed temperature was 600 °C to achieve a single-phase TiVO_4_ photoanode. The TEM analyses signify that the homogeneous distribution of TiVO_4_ nanoparticles' agglomeration formed a dendritic outer edge fashion. The highest photocurrent density at 1.8 V *vs.* RHE of TiVO_4_ was recorded at 400 μA cm^−2^ for the substrate temperature at 250 °C. Additionally, a narrower band gap (2.18 eV) of n-type semiconductor TiVO_4_ makes it a beneficial light absorber, thus driving PEC water splitting for hydrogen generation. These results further inspire TiVO_4_ employment as a photoanode material with a particular emphasis on their applications in PEC solar water splitting under 1 SUN.

Furthermore, sprayed TiVO_4_ photoanode showed excellent stability up to 6000 s. Besides, the photocurrent density monotonically improved from 73 to 400 μA cm^−2^ at 1.8 V *vs.* RHE with an increase in annealing temperature from 500 to 600 °C. Thus, the underlying photo-absorbing semiconductors could be fulfilled the lack of efficient photoanodes for the water-splitting reactions while not compromising either material's performance and achieving long-term passivation.

## Conflicts of interest

There are no conflicts to declare.

## Supplementary Material

RA-012-D2RA05894D-s001
