# Supplementary material for: Fabrication of TiVO4 photoelectrode for photoelectrochemical application
Source: RSC Adv. 2022 Dec 2;12(53):34640–51. doi: 10.1039/d2ra05894d (PMC9717350; doi:10.1039/d2ra05894d)
Supplement: RA-012-D2RA05894D-s001 [file RA-012-D2RA05894D-s001.pdf]

Electronic Supplementary Material (ESI) for RSC Advances.
This journal is © The Royal Society of Chemistry 2022

Supporting Information

Fabrication of $TiVO_4$ photoelectrode for photoelectrochemical application

Manal Alruwaili[1,2]*, Anurag Roy[1] , Srijita Nandy[1], Asif Ali Tahir[1]*

[1]Environment and Sustainability Institute, Faculty of Environment, Science and Economy, University of Exeter, Penryn TR10 9FE, United Kingdom.

[2] Physics Department, Faculty of Science, Jouf University, P.O. Box 2014, Sakaka 42421, Saudi Arabia

*Corresponding authors: ma942@exeter.ac.uk (M.A.); a.tahir@exeter.ac.uk (A.A.T.)

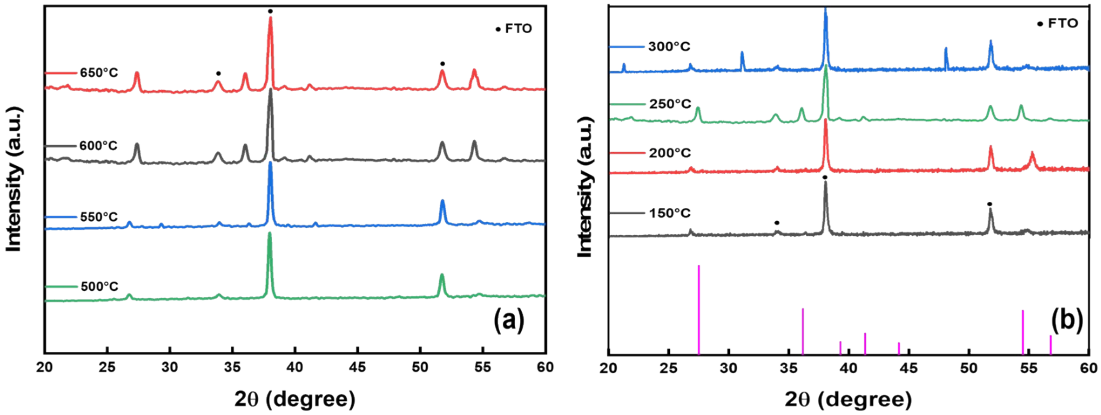

Fig. S1 XRD pattern of spray pyrolyzed $TiVO_4$ photoanode deposited on FTO glass at different annealing temperatures (a), and at different substrate temperatures (b).

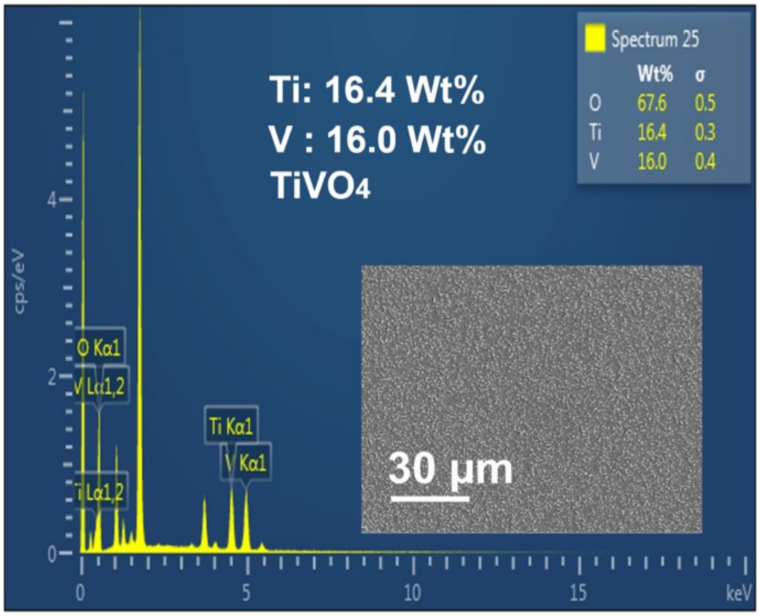

Fig. S2 EDS spectrum of pyrolyzed $TiVO_4$ photoanode deposited at 250°C.
